# Eye exercises of acupoints: their impact on myopia and visual symptoms in Chinese rural children

**DOI:** 10.1186/s12906-016-1289-4

**Published:** 2016-09-06

**Authors:** Zhong Lin, Balamurali Vasudevan, Su Jie Fang, Vishal Jhanji, Guang Yun Mao, Wei Han, Tie Ying Gao, Kenneth J. Ciuffreda, Yuan Bo Liang

**Affiliations:** 1 The Eye Hospital, School of Ophthalmology and Optometry, Wenzhou Medical University, No. 270 West College Road, Wenzhou, Zhejiang 325027 China; 2 College of Optometry, Mid Western University, Glendale, AZ USA; 3 Handan Eye Hospital, Handan, Hebei China; 4 School of Environmental Science &Public Health, Wenzhou Medical University, Wenzhou, Zhejiang China; 5 Department of Ophthalmology and Visual Sciences, The Chinese University of Hong Kong, Hong Kong, China; 6 Department of Biological and Vision Sciences, SUNY College of Optometry, New York, NY USA

**Keywords:** Eye exercises, Acupoints, Myopia, Near vision symptoms, CISS

## Abstract

**Background:**

Chinese traditional “eye exercises of acupoints” have been advocated as a compulsory measure to reduce visual symptoms, as well as to retard the development of refractive error, among Chinese students for decades. The exercises are comprised of a 5-min, bilateral eye acupoint self-massage. This study evaluated the possible effect of these eye exercises among Chinese rural students.

**Methods:**

Eight hundred thirty-six students (437 males, 52.3 %), aged 10.6 ± 2.5 (range 6–17) years from the Handan Offspring Myopia Study (HOMS) who completed the eye exercises and vision questionnaire, the convergence insufficiency symptom survey (CISS) questionnaire, and had a cycloplegic refraction were included in this study.

**Results:**

121 (14.5 %) students (64 males, 52.9 %) performed the eye exercises of acupoints in school. The multiple odds ratio (OR) and 95 % confidence interval (CI) for those having a “serious attitude” towards performing the eye exercises (0.12, 0.03–0.49) demonstrated a protective effect for myopia, after adjusting for the children’s age, gender, average parental refractive error, and the time spent on near work and outdoor activity. The more frequently, and the more seriously, the students performed the eye exercises each week, the less likely was their chance of being myopic (OR, 95 % CI: 0.17, 0.03–0.99), after adjusting for the same confounders. However, neither the “seriousness of attitude” of performing the eye exercises (multiple *β* coefficients: -1.58, *p* = 0.23), nor other related aspects of these eye exercises, were found to be associated with the CISS score in this sample.

**Conclusions:**

The traditional eye exercises of acupoints appeared to have a modest protective effect on myopia among these Chinese rural students aged 6–17 years. However, no association between the eye exercises and near vision symptoms was found.

**Electronic supplementary material:**

The online version of this article (doi:10.1186/s12906-016-1289-4) contains supplementary material, which is available to authorized users.

## Background

The traditional Chinese “eye exercises of acupoints” have been a compulsory measure performed by school children twice a day (5 min each morning and afternoon) for the purpose of relieving visual symptoms and reduction of myopia since the early 1960s. As described in detail previously in the Beijing Myopia Progression Study (BMPS), [1] they comprise bilateral acupoint self-massage that includes: (1) knead Tianying (Ashi) point, (2) press and squeeze Jingming (BL1), (3) press and knead Sibai (ST2), and (4) press Taiyang (EX-HN5) and scrape Cuanzhu (BL2), Yuyao (EX-HN4), Sizhukong (TE23), Tongziliao (GB1), Chengqi (ST1). Despite insisting on performing this intervention for overhalfa century, the prevalence of myopia and myopia-related visual impairment is on the rise in both urban and rural Chinese children [2–4]. Our previous study from BMPS found that the urban students who performed the eye exercises seriously, followed the instruction when performing the eye exercises, and were acquainted with these eye exercises, tended to have a lower convergence insufficiency symptom survey (CISS) score, e.g., less ocular-based, near fatigue symptoms [1]. However, the exercises appeared to have no measurable effect on the refractive error [1]. This could be related to the greater myopic refraction and apparent myopigenic environment among the urban students [1]. Hence, it would be interesting to determine if these eye exercises have an effect on myopia reduction in a parallel rural population with its less myopigenic environment.

The Handan Offspring Myopia Study (HOMS) was designed to determine the prevalence of myopia among rural children, namely the offspring of the Handan Eye Study (HES) population [5, 6]. It is noteworthy that the children from HOMS were in a similar age range (6–17 years), and had several vision examinations (e.g., visual acuity, ocular biometry, cycloplegic autorefraction) and questionnaires in common with the BMPS [6, 7]. Hence, the present study aimed to evaluate the impact of the eye exercises of acupoints among Chinese *rural* students, and furthermore to compare them to Chinese *urban* students.

## Methods

### Subjects

Details of the study design, sample size estimation, and baseline characteristics of HOMS were reported elsewhere [6]. Briefly, between October 2006 and October 2007, a population-based eye study in adults aged 30 years and older in Handan (Handan Eye Study, HES), Hebei province of North China, was conducted [5]. All participants (aged 6–17 years) along with at least one of their parents recruited in HES were included in HOMS between March 2010 and June 2010. Adopted children or children who had moved outside the county at least 6 months prior to this study were excluded. Finally, 878 (70.2 %) of 1238 were recruited [6]. The study followed the tenets of the Declaration of Helsinki, and it was approved by the Ethics Committee of the Handan Eye Hospital. Written, informed-consent was obtained from the children’s parents/guardians. In the current study, 836 students who had completed the eye exercises of acupoints questionnaire, the standard CISS questionnaire, and had a cycloplegic refraction were included. Of these, 121 (14.5 %) performed the eye exercises of acupoints in school.

### Activity questionnaire

The activity questionnaire used in the Sydney Myopia Study was translated into Chinese with minor modifications [8, 9]. This questionnaire included components such as duration of near and far work, living environment, eating habits, and general health. These activities were grouped into near work and outdoor activities. Details of the activity questionnaire were reported elsewhere [9, 10].

### Eye exercises of acupoints questionnaire

The eye exercises of acupoints were performed twice a day (morning and afternoon), each time for 5 continuous minutes in each school day. The participants were also asked to complete an acupoints eye exercise questionnaire. It consisted of 11 items related to motivation, frequency, and attitude towards the eye exercises of acupoints. Details of the eye exercises of acupoints were reported elsewhere [1] (Additional file 1).

### Convergence insufficiency symptom survey (CISS)

The CISS consists of 15 items with 5 response categories for each item [11]. It is scored as never (0), infrequently (1), sometimes (2), fairly often (3), and always (4). It covered reading and other near work activities. The total score is obtained as a sum of scores for all 15 items (range from 0 to 60) (Additional file 2).

### Refractive error

All students received a cycloplegic autorefraction (*KR8800, Topcon, Tokyo, Japan*), whereas the parents received non-cycloplegic autorefraction due to their age and related reduced accommodation. Cycloplegic autorefraction was performed 20 min after instilling 3 drops of cyclopentolate 1 % (Cyclogyl, Alcon; Fort Worth, TX, USA). Three readings of refractive error were obtained and averaged for further analysis for each eye in all participants.

### Data analysis

Due to the high correlation of the cycloplegic refractive error (spherical equivalent, SE = Spherical refraction + ½ cylindrical refraction) between the right and left eyes (Pearson correlation coefficient 0.96, *p* < 0.001), only the SE of the right eye of each student was used in the analysis. Myopia was defined as SE ≤ -0.50D [1, 7]. Parental refractive error was defined as the average of the non-cycloplegic SE of each eye of the father and mother combined.

Both univariate and multiple (after adjusting for putative risk factors for myopia, e.g., children’s age, gender, average parental refractive error, time spent on near work and outdoor activity) odds ratio (OR) and the 95 % confidence interval (CI) for myopia for different items of the eye exercises of acupoints question were calculated using generalized linear models (GLMs). Univariate and multiple (adjusting for the same confounding factors) regression analyses were performed for the CISS score with the different items of the eye exercises of acupoints using GLMs.

## Results

A total of 836 students (437 males, 52.3 %) with a completed acupoints eye exercise questionnaire, completed convergence insufficiency symptom survey questionnaire (CISS), and a cycloplegic refraction were included in the current analysis. Of these, 121 (14.5 %) students (64 males, 52.9 %) performed the eye exercises of acupoints in school (Table 1). Students who performed the eye exercise in school were older (11.8 ± 2.3 years vs. 10.3 ± 2.4 years, *p* < 0.001), more myopic (-0.40 ± 1.62 D vs. 0.06 ± 1.31 D, *p* = 0.004), and had a higher CISS score (14.3 ± 6.4 vs. 10.7 ± 6.8, *p* < 0.001) compared to those who did not.

**Table 1:** Demographic characteristics of children who performed eye exercises and those who did not performed eye exercises of acupoints in the Handan Offspring Myopia Study

	Children performed eye exercises (*n* = 121)	Children did not perform eye exercises (*n* = 715)
Age, years	11.8 ± 2.3	10.3 ± 2.4
Gender, male/female	64/57	373/342
Height, cm	146.3 ± 12.5	138.5 ± 13.5
Weight, kg	39.2 ± 10.4	33.4 ± 10.2
Cycloplegic SE, diopter	−0.40 ± 1.62	0.06 ± 1.31
Myopia, number (%)	50 (41.3)	148 (20.7)
Paternal average SE, diopter	−0.46 ± 0.69	−0.54 ± 0.77
CISS score	14.3 ± 6.4	10.7 ± 6.8

*SE* spherical equivalent, *CISS* convergence insufficiency symptom survey

Table 2 summarizes the distribution of students’ responses against each item of the eye exercises of acupoints questionnaire. It also presents the student’s SE, and the univariate and multiple OR for myopia, for each item of the eye exercises questionnaire. Although students who performed the eye exercises in school were more myopic compared to those who did not (-0.40 ± 1.62 D vs. 0.06 ± 1.31 D, *p* = 0.004), performing the eye exercises in school did not reveal a significant effect for myopia per se (multiple OR, 95 % CI: 1.97, 1.19–3.26). Those who performed the eye exercises seriously demonstrated a borderline protective effect for myopia (univariate OR, 95 % CI: 0.46, 0.20–1.05), that is, less myopia. However, this protective effect became *significant* after adjusting for the student’s age, gender, average parental refractive error, and time spent on near work and outdoor activity (OR, 95 % CI: 0.12, 0.03–0.49). Moreover, in comparison to students who never performed the eye exercises, those who performed them seriously less than 3 times per week (univariate OR, 95 % CI: 0.41, 0.18–0.93), and every time per week (univariate OR, 95 % CI: 0.28, 0.09–0.87), had less chance of being a myope. Furthermore, after adjusting for the same confounders, students who performed the eye exercises of acupoints seriously each time per week had less chance of being a myope (OR, 95 % CI: 0.17, 0.03–0.99). No other significant effects were observed.

**Table 2:** Children’s spherical equivalent (SE) and odds ratio (OR) for myopia for each item of the eye exercises of acupoints questionnaire

	Number (%)	SE (mean ± SD)	Univariate OR (95 % CI)	Multiple OR (95 % CI)^a^
Performed the eye exercises (in school)				
No	715 (85.5)	0.06 ± 1.31		
Yes	121 (14.5)	−0.40 ± 1.62^b^	2.70 (1.80, 4.04)	1.97 (1.19, 3.26)
Times per day (in school)				
< 2	101 (83.5)	−0.45 ± 1.67		
≥ 2	20 (16.5)	−0.14 ± 1.35	1.20 (0.46, 3.15)	0.78 (0.22, 2.76)
Serious or not				
No/ moderate	83 (68.6)	−0.52 ± 1.80		
Yes	38 (31.4)	−0.13 ± 1.12	0.46 (0.20, 1.05)	0.12 (0.03, 0.49)
Serious times per week				
None	53 (43.8)	−0.77 ± 1.81		
< 3	48 (39.7)	−0.12 ± 1.56	0.41 (0.18, 0.93)	0.50 (0.18, 1.41)
Every time	20 (16.5)	−0.09 ± 0.97	0.28 (0.09, 0.87)	0.17 (0.03, 0.99)
Eye exercises were taught by				
Atlas/ classmate	47 (38.8)	−0.65 ± 1.59		
Teacher/doctor/health counselor	74 (61.2)	−0.25 ± 1.64	0.66 (0.32, 1.40)	0.53 (0.20, 1.44)
Speed				
Faster/slower than the broadcast & at will	88 (72.7)	−0.29 ± 1.52		
Following the broadcast	33 (27.3)	−0.68 ± 1.88	1.26 (0.56, 2.83)	1.63 (0.52, 5.13)
Acupoints acquaintance				
No/moderate	106 (87.6)	−0.40 ± 1.67		
Yes	15 (12.4)	−0.37 ± 1.25	0.94 (0.31, 2.83)	1.09 (0.21, 5.78)
Perform additional eye exercises (outside school)				
No	95 (78.5)	−0.39 ± 1.66		
Yes	26 (21.5)	−0.43 ± 1.53	1.57 (0.66, 3.75)	1.36 (0.39, 4.67)

*SE* spherical equivalent, *SD* standard deviation, *OR* odds ratio, *CI* confidence interval; the first group was the reference group

Multiple OR ^a^adjusted for children’s age, gender, average parental refractive error, times spent on near work and outdoor

^b^significantly different compared to the first group

When regression analysis was performed using the children’s SE as the dependent variable and items from the eye exercises of acupoints questionnaire as the independent variable, similar results were found. The more often students performed the eye exercises of acupoints per week, the less myopic SE the students had (univariate *β =* 0.40, *p* = 0.047). After adjusting for the students’ age, gender, average parental refractive error, and time spent on near work and outdoor activity, those with a serious attitude for performing them (multiple *β =* 0.73, *p* = 0.043), and with a higher frequency of performing them seriously, still remained borderline significant (multiple *β =* 0.44, *p* = 0.050).

Table 3 presents the CISS score, as well as the univariate and multiple *β* coefficients of the CISS score, for each item of the eye exercises questionnaire. Students who performed the eye exercises of acupoints in school had a higher CISS score (14.3 ± 6.4 vs. 10.7 ± 6.8, *p* < 0.001) compared to those who did not, and this trend remained significant after adjusting for the student's age, gender, average parental refractive error, and time spent on near work and outdoor activity (multiple *β =* 1.95, *p* = 0.005). However, no other items related to the eye exercises of acupoints, including a seriousness of attitude of performing them (multiple *β* = -1.58, *p* = 0.23), and acupoints acquaintance (multiple *β* = 0.90, *p* = 0.67), were found to have an effect on the CISS score; that is, there was no significant effect on relieving the near vision symptoms, in these students. Similar results were found when the student’s refractive error was further adjusted.

**Table 3:** Children’s convergence insufficiency symptom survey scores(CISS) and *β* coefficientsforeach item of the eye exercises of acupoints questionnaire

	CISS score (mean ± SD)	Univariate *β* coefficient (*p* value)	Model 1^a^	Model 2^b^
Performed the eye exercises (in school)				
No	10.7 ± 6.8			
Yes	14.3 ± 6.4^c^	3.63 (<0.001)	1.95 (0.005)	1.88 (0.006)
Times per day (in school)				
< 2	14.1 ± 6.4			
≥ 2	15.4 ± 6.0	1.31 (0.40)	2.30 (0.13)	2.19 (0.15)
Serious or not				
No/moderate	14.6 ± 6.4			
Yes	13.7 ± 6.4	−0.94 (0.45)	−1.58 (0.23)	−1.88 (0.16)
Serious times per week				
None	13.9 ± 6.1			
< 3	15.2 ± 6.3			
Every time	13.1 ± 7.3	−0.06 (0.94)	0.25 (0.76)	0.12 (0.89)
Eye exercises were taught by				
Atlas/classmate	15.7 ± 5.4			
Teacher/doctor/health counselor	13.6 ± 6.8	−2.12 (0.08)	−0.84 (0.49)	−1.00 (0.42)
Speed				
Faster/slower than the broadcast & at will	13.7 ± 6.2			
Following the broadcast	15.9 ± 6.7	2.25 (0.08)	1.43 (0.31)	1.65 (0.25)
Acupoints acquaintance				
No/moderate	14.2 ± 6.2			
Yes	15.3 ± 6.0	1.17 (0.51)	0.90 (0.67)	0.89 (0.67)
Perform additional eye exercises (outside school)				
No	14.1 ± 6.7			
Yes	15.1 ± 5.2	0.98 (0.49)	1.96 (0.18)	1.95 (0.19)

*CISS* convergence insufficiency symptom survey, *SD* standard deviation

Model 1 ^a^adjusted for children’s age, gender, average parental refractive error, times spent on near work and outdoor

Model 2 ^b^adjusted for Model 1 + children’s refractive error

^c^significantly different compared to the first group

## Discussion

The rural students who performed the eye exercises of acupoints in school were more myopic, and furthermore they had a higher CISS score as compared to those who did not. There could be two possible reasons for this outcome. First, students with a more myopic refractive error might be more determined, or under greater psychological pressure, to perform the eye exercises of acupoints, to stabilize their myopia and to prevent visual symptoms. Second, students who performed these eye exercises in school had a more intense near work load than those who did not (5.43 ± 2.01 vs. 4.66 ± 1.53 h per day, *p* < 0.001).

There were several interesting and important findings in this study, which differed from our Chinese urban study [1]. First, although the eye exercises of acupoints were compulsory in all Chinese school children, only approximately 15 % of the rural students actually performed them in school, much less than the 96.6 % among the urban students [1]. Second, there was no association with the CISS score, e.g., ocular-based vision symptoms, and any item of the acupoints eye exercises questionnaire. Third, and most importantly, the more frequently the students performed the eye exercises seriously, the less myopic refractive error they had, which suggested a protective effect for myopia, even after adjusting for possible confounders.

Several Chinese studies have reported on the eye exercises of acupoints and juvenile myopia. One epidemiological study (*n* = 612) reported that the prevalence of myopia was lower in grade 2–6 primary school children who performed the eye exercises regularly, as compared to children who performed them infrequently (29.53 % vs. 38.52 %) [12]. Another study demonstrated that these eye exercises were protective for juvenile myopia [13]. It has also been reported that having a “serious attitude” towards performing the eye exercises improved visual acuity in grades 1–2 primary school children [14]. However, the underlying mechanism of these eye exercise to reduce myopia remains unclear. One study indicated that they could increase the peak systolic velocity (PSV) in the central retinal and ophthalmic arteries, and thus reduce their resistance index (RI), as observed by color Doppler imaging [15]. In addition, simple cessation of near work to perform the eye exercises provides a short rest period that itself may reduce the visual symptoms [16].

In our previous urban study (BMPS), less myopic refractive error was observed in students who performed the eye exercises of acupoints seriously. However, the protective effect of these eye exercises for myopia was not significant after adjusting for the students’ age, gender, parental refractive error, and time spent doing near work and outdoor activity. More importantly, students who performed the eye exercises seriously, followed the instructions when performing the eye exercises, and were acquainted with the eye exercises, tended to have a lower CISS score, i.e., were less symptomatic when performing near work activities, even after adjusting for the same confounders [1].

Convergence insufficiency is associated with visual symptoms at near, including general eyestrain, blurred vision, diplopia, difficulty concentrating, and reduced comprehension after short periods of reading or performing or other near activities [11, 17, 18]. Studies have demonstrated that the CISS questionnaire is a valid instrument for quantifying near visual symptoms in 9 to 18 year-old children and teenagers [11, 19]. In the present study, unlike the previous urban sample, [1] no association between seriousness of attitude of performing eye exercises of acupoints (multiple *β* = -1.58, *p* = 0.23), or acupoints acquaintance (multiple *β* = 0.90, *p* = 0.67), and near vision symptoms was found. Due to the correlation between accommodation, vergence, and refractive error, [20] a further multiple regression model with the refractive error adjusted was performed, which yielded similar results.

Consistent with previous studies on the eye exercises of acupoints published in the Chinese literature, [12–14] but different from our previous studies on urban students, [1] rural students in the present study who performed the eye exercises seriously tended to have less change in their myopia. This could be due to a dose-effect of the eye exercises of acupoints for myopia. In the current study, the rural students who performed the eye exercises in school had less myopic refractive error as compared to the urban students (-0.40D vs. -1.70D). Moreover, as compared to urban students, the rural students are exposed to relatively low risk factors for myopia, such as spending less time on near work and more time on outdoor activities, having a more open and spacious living environment, and having fewer myopic parents [21–24]. Lastly, urban student’s myopia and related near oculomotor imbalance may be more “embedded” in those with intensive near work demands, and thus less susceptible to any remediation/intervention [25]. Thus, the effect of these daily eye exercises on prevention of myopia for 10 min each day may manifest an effect in the rural, but not in the urban, school students. Also, and again different from the urban students, the eye exercises were not associated with relieving ocular-based visual symptoms.

There were some possible limitations to the present study. First, the two subgroups of students, i.e., those participants versus non-participants in performing the eye exercises at school, were somewhat heterogeneous. The students who performed the eye exercises of acupoints in school were older, more myopic, and had a higher CISS score as compared to those who did not. Second, there was only a relatively small sample of students who actually performed the eye exercises in school. This may have reduced the power to uncover additional associations, e.g., with the CISS score. Third, there may be recall bias, since the questionnaires were used for collecting the information for eye exercises of acupoints, as well as other information (e.g., activities). Fourth, cross-sectional data cannot provide direct evidence on the association between the eye exercises and myopia development. Moreover, the results of this study would be stronger with either a control or additional comparative group. Hence, a randomized controlled trial with a larger sample size, and perhaps different “doses” of acupoints eye exercise schedules, is warranted to understand better the possible effect of eye exercises of acupoints on myopia and related near vision symptomatology.

## Conclusions

This cross-sectional study found that the traditional eye exercises of acupoints had a modest protective effect on myopia among these Chinese rural students aged 6–17 years. However, no association between the eye exercises and near vision symptoms was revealed.

## Additional files

Additional file 1: Eye exercises of acupoints questionnaire. (DOCX 12 kb) Additional file 2: Convergence insufficiency symptom survey. (DOCX 13 kb) Additional file 3: Dataset. (SAV 53 kb)

## Abbreviations

BMPS: The Beijing Myopia Progression Study
CI: Confidence interval
CISS: Convergence insufficiency symptom survey
HES: The Handan Eye Study
HOMS: The Handan Offspring Myopia Study
OR: Odds ratio
