# Supplementary material for: Eye exercises of acupoints: their impact on myopia and visual symptoms in Chinese rural children
Source: BMC Complement Altern Med. 2016 Sep 6;16(1):349. doi: 10.1186/s12906-016-1289-4 (PMC5013592; doi:10.1186/s12906-016-1289-4)
Supplement: Additional file 1: — Eye exercises of acupoints questionnaire. (DOCX 12 kb) [file 12906_2016_1289_MOESM1_ESM.docx]

**EYE EXERCISES OF ACUPOINTS QUESTIONNAIRE**

1. Do you perform eye exercises of acupoints in school?

□Yes □No

2. What is your reason for performing eye exercises of acupoints?

□Relieving ocular fatigue □Required by teachers or parents

□Other reason, please specify: _______________________

3. How often do you perform eye exercises of acupoints in school?

□More than twice a day □Twice a day □Once a day □Less than once a day

4. Do you think you perform eye exercises of acupoints seriously?

□Yes □Moderate □No

5. How many times do you perform eye exercises of acupoints seriously per week?

□None □Less than 3 times □More than 5 times □Every time

6. Who taught you how to perform eye exercises of acupoints?

□School teacher(s) □Learn from health atlas

□Doctor(s) or school doctor(s) □Learn from classmate(s)

7. How fast do you perform eye exercises of acupoints?

□Faster than the broadcast □Follow the broadcast

□Slower than the broadcast □At will

8. Are you acquainted with the acupoints of eye exercises of acupoints?

□Yes □Moderate □No

9. Do you perform eye exercises of acupoints outside the school hours?

□Yes □No (completed the questionnaire here)

10. How often do you perform eye exercises of acupoints outside the school hours?

□More than twice a day □Twice a day □Once a day □Less than once a day

11. What is your reason of performing eye exercises of acupoints outside the school hours?

□Relieving ocular fatigue □Required by teachers or parents

□Other reasons, please specify: _______________________
