# Supplementary material for: Eye exercises of acupoints: their impact on myopia and visual symptoms in Chinese rural children
Source: BMC Complement Altern Med. 2016 Sep 6;16(1):349. doi: 10.1186/s12906-016-1289-4 (PMC5013592; doi:10.1186/s12906-016-1289-4)
Supplement: Additional file 2: — Convergence insufficiency symptom survey. (DOCX 13 kb) [file 12906_2016_1289_MOESM2_ESM.docx]

**CONVERGENCE INSUFFICIENCY SYMPTOM SURVEY**

|  |  | **Never** | **Infrequently** | **Sometimes** | **Fairly often** | **Always** |
| --- | --- | --- | --- | --- | --- | --- |
| 1. | Do your eyes feel tired when reading or doing close work? |  |  |  |  |  |
| 2. | Do your eyes feel uncomfortable when reading or doing close work? |  |  |  |  |  |
| 3. | Do you have headaches when reading or doing close work? |  |  |  |  |  |
| 4. | Do you feel sleepy when reading or doing close work? |  |  |  |  |  |
| 5. | Do you lose concentration when reading or doing close work? |  |  |  |  |  |
| 6. | Do you have trouble remembering what you have read? |  |  |  |  |  |
| 7. | Do you have double vision when reading or doing close work? |  |  |  |  |  |
| 8. | Do you see the words move, jump, swim or appear to float on the page when reading or doing close work? |  |  |  |  |  |
| 9. | Do you feel like you read slowly? |  |  |  |  |  |
| 10. | Do your eyes ever hurt when reading or doing close work? |  |  |  |  |  |
| 11. | Do your eyes ever feel sore when reading or doing close work? |  |  |  |  |  |
| 12. | Do you feel a "pulling" feeling around your eyes when reading or doing close work? |  |  |  |  |  |
| 13. | Do you notice the words blurring or coming in and out of focus when reading or doing close work? |  |  |  |  |  |
| 14. | Do you lose your place while reading or doing close work? |  |  |  |  |  |
| 15. | Do you have to re-read the same line of words when reading? |  |  |  |  |  |
|  |  | x 0 | x 1 | x 2 | x 3 | x 4 |

**TOTAL SCORE**______________
